# Supplementary figures and images for: Identification of potential genes in upper tract urothelial carcinoma using next-generation sequencing with bioinformatics and in vitro analyses
Source: PeerJ. 2021 Apr 27;9:e11343. doi: 10.7717/peerj.11343 (PMC8086570; doi:10.7717/peerj.11343)

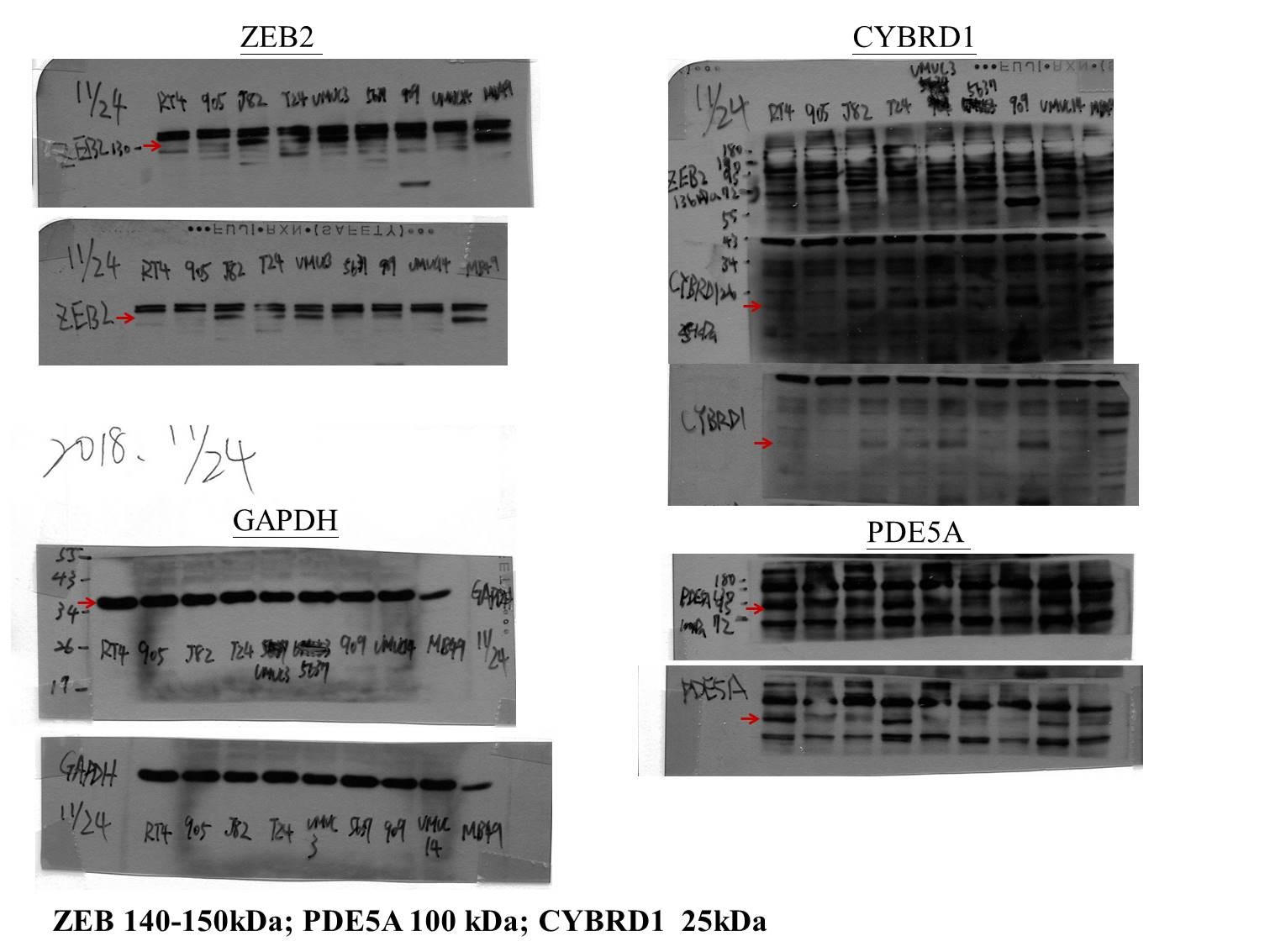

Supplement: Supplemental Information 3 [file peerj-09-11343-s003.jpg]
